# High-Precision Thin Wall Bipolar Plates for Fuel Cell Applications via Injection Compression Molding with Dynamic Mold Temperature Control

**DOI:** 10.3390/polym14142799

**Published:** 2022-07-08

**Authors:** Benedikt Roth, Rainer Frank, Tobias Kleffel, Kevin Schneider, Dietmar Drummer

**Affiliations:** Institute of Polymer Technology, Friedrich-Alexander-Universität Erlangen-Nürnberg (FAU), Am Weichselgarten 10, 91058 Erlangen, Germany; rainer.frank@fau.de (R.F.); tobias.kleffel@fau.de (T.K.); kevin.s.schneider@fau.de (K.S.); dietmar.drummer@fau.de (D.D.)

**Keywords:** injection compression molding, bipolar plate, fuel cell, thin-wall injection molding, dynamic mold temperature control

## Abstract

In recent years, the demand for polymer compound solutions for the application of bipolar plates in polymer electrolyte membrane fuel cells (PEMFC) has increased continuously due to significant cost and lifetime advantages compared to metallic solutions. The main challenge of the compounds is the high filler content required to ensure sufficient electrical conductivity of the bipolar plates. The associated increase in viscosity and simultaneously increased thermal conductivity limit the conventional injection molding process in terms of achievable flow path length to wall thickness ratios (plate aspect ratio). In order to evaluate the extent to which highly modified electrically conductive polymer material systems can be processed into thin-walled and highly dimensionally stable bipolar plates, an injection compression molding process with dynamic mold temperature control (ICM-DT) has been developed. For this purpose, a compound was prepared from polypropylene (PP) and graphite-flakes. The compound was characterized with respect to the achieved filler content, the electrical conductivity, as well as the pressure- and temperature-dependent solidification range. The ICM-DT experiments were carried out by varying the maximum mold temperature and the compression force. In addition, the process was designed with multiple compression and decompression steps to account for a possible pressure-dependent solidification of the compound. The plates were characterized with respect to the achieved plate aspect ratio and the flow-path-dependent dimensional thickness stability. It was shown that the plate aspect ratio could be increased by up to 125% with the maximum filler content compared to conventional injection molding processes provided in the literature. With the multi-stage ICM-DT process, it was also possible to optimize the thickness dimensional stability with a maximum deviation of 3% over the flow path.

## 1. Introduction

In the course of the energy system transformation and the electrification of the transport sector, emission-free drive and storage concepts are becoming increasingly important. In this context, the technology of the polymer electrolyte membrane fuel cell (PEMFC) plays an important role in the commercial use of hydrogen as a possible energy storage medium for mid- and long-distance concepts in the transport sector (truck, marine, and aviation) as well as stationary and decentralized energy supply in the energy sector [1]. However, to make this technology usable and economically attractive for broad application areas, the significant optimization of the total cost and lifetime of the fuel cell compared to fossil fuel drive and storage concepts is required [2]. Ideally, the optimization of the stack design and construction of the individual components should be performed in a manner that improves the performance and lifetime of the cell [3] while keeping the thicknesses and volumes of the individual components as low as possible for cost and space reasons [4]. In addition to the design constraints, high demands are placed on the underlying materials. Along the process chain, the bipolar plate shows the most pronounced requirement profile and optimization potential, since it causes 30–40% of the total costs [5] and up to 80% of the total weight of a fuel cell [6]. Thus, high electrical conductivities, sufficient mechanical stability, and high corrosion resistance are required, along with low weight and installation space volume, low costs, and optimized replication of channel structures for distributing the process gases [2]. To meet this requirement profile, research is currently being conducted on both metallic materials and polymer compound solutions. Although metallic materials allow high electrical conductivities while maintaining high mechanical stability, flexibility [7], and low component thickness [8], they are corroded and oxidized by the reaction products released during the electrochemical reaction [9]. The consequences are a deterioration in power output due to increased contact resistance and a reduced lifetime of the cell [10], which is why expensive and complex corrosion coatings are usually applied [4]. Furthermore, the fabrication processes of metallic bipolar plates are limited either in terms of efficiency and process costs [11] or in terms of the complexity of the channel structures such that excessively high channel aspect ratios often cause dimensional errors as well as cracks and wrinkles due to the excessive thinning of the substrate [12]. In contrast, polymer compound-based bipolar plates offer an increasingly attractive technical and economic alternative to metal solutions due to significant advantages such as reduced specific weight, good corrosion resistance [13], durability [10], high functional integration within complex 3D geometries, and a low price [14].

The electrical insulation effect of polymers can be significantly influenced by fillers and additives mixed into the polymer matrix. By adding conductive fibers, flakes, or spheres consisting of, e.g., metals, conductive carbon black, graphite, and nanofillers such as carbon nanotubes (CNT), the resistivity of the polymers can be reduced by several orders of magnitude [15]. Multiple studies were carried out to achieve high electrical conductivity of bipolar plates by different filler modifications. For example, mixtures of conductive carbon black and graphite powder (total filler content: 80 wt.%) [16] or conductive carbon black and carbon microbeads (total filler content: 80 wt.%) [17], each in 2 mm thick polyphenylene sulfide (PPS) plates, were able to achieve volume resistivities of 10^−2^ Ω·cm. In addition, Rzeczkowski et al. [18] investigated the effect of adding expanded graphite to a polypropylene (PP) matrix on the resulting electrical conductivity. They were able to achieve a reduction in the average volume resistivity of about 1600% up to 15.9 S·cm^−1^ by increasing the filler content from 10 to 80 wt.% in 2 mm thick plates. The underlying mechanism of the decrease in electrical resistance is the sufficient contact and interaction of the particles and fillers with each other so that they can form a conductive percolation network [19]. Numerous studies have shown that the level of electrical conductivity is directly proportional to the number of contact points of the individual filler particles and thus strongly dependent on the filler content [20,21,22]. The filler content at which a percolation path is formed is called the percolation threshold and is strongly dependent on the filler’s shape and size [15]. For example, platelets or rod-shaped fillers with a high aspect ratio led to comparatively low percolation thresholds than spherical fillers [23,24]. In addition, due to the higher probability of mutual interaction, the percolation threshold was reached at lower filler contents for smaller particles compared to larger fillers. This results in improved electrical conductivities with small particles of high aspect ratios, especially at higher filler contents. Investigations on the influence of filler shape and size on the electrical conductivity of highly filled compounds of PP and graphite (total filler content: 78 wt.%) for fuel cell applications were able to demonstrate a conductivity reduction in spherical particles by a factor of 1.5 compared to platelet-shaped particles of the same size. In addition, 5 μm platelets achieved a conductivity up to 2.5 times higher than 20 μm platelets with the same filler content [25].

The viscosity of a polymer melt is a function of the molecular structure of the macromolecules and is determined by the interaction of the polymer chains with each other. In addition to the basic structural composition of the polymer (chain length/chain branching), this is largely determined by the temperature [15], pressure [26], and shear conditions [27] during the filling process in the injection molding process. Furthermore, the addition of fillers results in significantly changed flow behavior due to flow restrictions caused by the interaction between the polymer matrix and the filler particles, or by interaction and agglomeration of the fillers with each other [28]. Thus, the viscosity of filled polymer melts shows a clear dependence on the volume fraction of the fillers, the particle shape, the average particle size and the particle size distribution [29]. Accordingly, higher volume fractions and higher aspect ratios (length/thickness) of the fillers lead to an increase in melt viscosity compared to the unfilled polymer [30]. For example, Mighri et al. [20] were able to determine an increase in viscosity by a factor of 3–10 as a function of shear rate for a filler content of 25 wt.% carbon black particles in a PP matrix compared to pure PP. Studies by Markov were also able to attribute a viscosity increase by a factor of 5 in a polyamide 6 (PA6) melt filled with anisometric copper platelets compared to isometric (spherical) particle geometry at the same filler content, particle size, and shear rate due to the specific surface area changing with the particle geometry [31]. Furthermore, the influence of particle size on the viscosity of 30 vol.% graphite-flake filled PA6 melts has been determined. At lower shear rates, viscosity increases strongly with decreasing particle size, whereas the effect of particle size vanishes at higher shear rates [31]. The high filler content of the modified compounds, required for sufficient electrical conductivity of the bipolar plates, results in limitations in the possible thickness dimensions of current plate geometries in the injection molding process. In recent studies, for example, plate dimensions of 80 × 80 × 2 mm^3^ [18] or 30 × 30 × 1.5 mm^3^ [6] have already led to considerable replication and filling problems in conventional injection molding.

In addition to the increased viscosity due to a high filler content, the temperature dependence of viscosity is, furthermore, negatively influenced by increased thermal conductivities of electrically modified polymer melts [32]. Due to the lack of free electrons in unfilled polymers, thermal energy can only be transferred in the form of elastic waves, so-called phonon oscillations, along the polymer chain or via van der Waals and hydrogen bonds (secondary valence forces) to neighboring chain segments. As a result of the presence of free mobile charge carriers in electrically conductive fillers, phonon vibrations can, thus, be additionally transported via the filler network [19]. This results in an accelerated heat transfer from the filled melt to the mold and, thus, further complicates the precise molding of fine channel structures and high aspect ratios due to higher cooling rates [6].

Components with small dimensions show significantly different time-temperature-pressure conditions during processing compared to macroscopic components. The increased surface-to-volume ratio (S/V) of micro and thin-walled components results in accelerated heat transfer between melt and mold [33]. Because of the faster cooling of the melt across the component cross-section, flow resistance increases due to the temperature-induced increase in viscosity. In addition, the rapidly solidified surface layers take up a significantly larger proportion of the total flow cross-section compared to macroscopic components. This often leads to filling and replication problems of fine surface structures [34]. Among other things, this is attempted to be compensated by high mold temperatures [35] or high injection speeds [36]. The latter, in combination with reduced flow cross sections, also results in an increased pressure requirement during filling [37]. For example, Yokoi et al. [38] were able to measure injection pressures of up to 3000 bar at an injection speed of up to 1000 mm·s^−1^ over a flow path of 150 mm at a plate thickness of 2 mm. The highest pressures occur close to the gating point with simultaneous high deformation of the polymer melt due to shear and elongation. With increasing distance to the gate, the flow velocity and, therefore, the shear and pressure influence decrease due to the high-pressure loss in the gate area. Investigations on thin-walled injection molded components have shown that the inhomogeneous pressure distribution during the filling process results in significant deviations in density [39] and thickness dimensions [40] along the flow path length, both with dynamic and conventional isothermal mold temperature control. The highest density and thickness values are measured close to the gate, since the polymer solidifies here under the full pressure effect, whereas, towards the end of the flow path, the polymer solidifies almost without pressure effect [41]. Since the melt viscosity exhibits a pronounced pressure dependence [42], the high injection pressures required in thin-wall molding further increase the viscosity and thereby intensified the effect of replication and dimensional stability problems.

In order to utilize the urgently needed cost and lifetime advantages of the polymers for the application of bipolar plates, high filler contents are necessary for achieving sufficiently high electrical conductivity. However, thermoplastic melts modified in this manner show deteriorated replication and mold filling behavior due to significantly increased viscosity and the negative effect of increased melt thermal conductivity. This leads to limitations in the possible thickness dimensions and dimensional stability of previous bipolar plate geometries in the injection molding process. A comparison with metallic bipolar plates, which can already be manufactured in thicknesses of down to 100 µm [8], illustrates that the specific weight advantage of the polymers cannot be utilized to date either. Since the output voltage per cell is restricted to a maximum of 1.23 V, several cells connected in series are required for higher voltages. This also results in considerably larger installation space volumes and costs for thicker bipolar plates made of polymer compounds. The objective of reducing the plate thickness cannot be achieved by higher injection speeds and mold temperatures sufficiently, since this results in excessive pressure requirements during filling [38] and inhomogeneous pressure and temperature fields [41] over the flow path. This is caused by the reduced flow cross-section, irrespective of the type of mold temperature control. At the component level, this is reflected in a deteriorated, flow-path-dependent dimensional thickness accuracy [40] and molding accuracy of the channel structures [6].

Therefore, the aim of this study is the development of an injection compression molding process with dynamic mold temperature control (ICM-DT) for the realization of thin-walled and highly filled bipolar plates with the highest possible thickness dimensional stability. A dynamic mold temperature control is intended to maintain the flowability of the polymer melt during the molding process. The combination of this strategy with a compression molding process allows a two-dimensional holding pressure effect and realization of high plate aspect ratios with the highest possible dimensional stability. In addition to characterizing the compound with regard to process-relevant solidification properties for analysing the ICM-DT process, electrical conductivity was determined as a function of the filler content. During ICM-DT experiments, the compound with maximum conductivity was used to determine the influence of mold temperature and compression pressure on the resulting dimensional stability of the plate thickness. In order to take into account a possible pressure-dependent solidification of the compound, a modified multi-stage ICM-DT process has been designed and optimized with regard to the achievable dimensional thickness stability and plate aspect ratio.

## 2. Materials, Specimen and Methods

### 2.1. Materials

For the experiments, flake-shaped graphite particles of the type GraphCOND (Georg H. LUH GmbH, Walluf, Germany) with a percentile value of D90: 50–70 µm was compounded at 50, 60, 70, and 80 wt.% proportions into a PP matrix type 505P (Sabic Corporation, Riyadh, Saudi Arabia) by means of a twin screw extruder type ZSE HP 27 (Leistritz Group, Nuremberg, Germany). Table 1 presents an overview of the main compounding parameters.

### 2.2. Specimen

**Figure 1:**
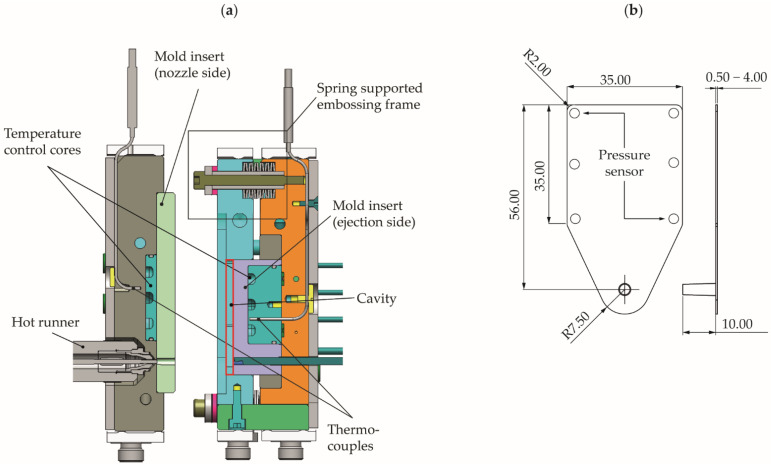
(**a**) Schematic representation of the injection mold; (**b**) plate specimen with the positions of the ejector pins and indirect pressure sensors behind two of them (all dimensions in mm).

For the investigation of electrical conductivity, plate aspect ratio, and dimensional accuracy, a plate specimen with variable plate thickness was used. The production using ICM-DT was carried out on an injection molding machine type Arburg Allrounder 370 U (Arburg GmbH + Co. KG, Lossburg, Germany). Figure 1 shows a schematic representation of the injection mold (a) and the dimensions of the plate specimens with marks of the positions of the ejector pins and indirect pressure sensors behind two of these (b).

The compression stroke was performed via the main axis of the machine by means of an embossing frame, which is supported by disc springs to the ejector-side mold plate, enabling a variable adjustable component thickness between 0.5 and 4 mm. The dynamic temperature control of the mold was carried out in the embossing frame as well as in the temperature control cores close to the cavity on the nozzle and ejector side in a double circuit of pressurized hot and cold water with a switchover unit close to the cavity. The mold has temperature sensors on the nozzle and ejector sides as well as two indirect cavity pressure sensors located behind the ejector pins at the positions near and far from the gate.

**Figure 2:**
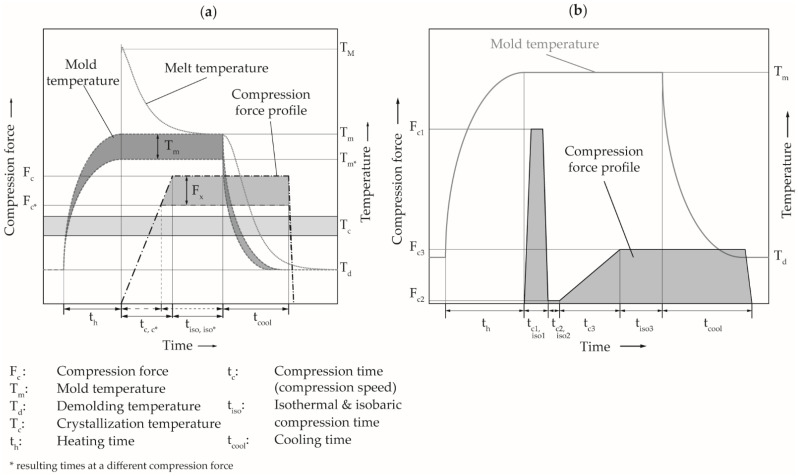
(**a**) Schematic process illustration of the production of the test specimens using ICM-DT; (**b**) schematic illustration of the multistage ICM-DT process.

The process cycle of the ICM-DT experiments is schematically shown in Figure 2a. First, the mold is heated to the investigated mold temperature. Afterwards, the melt is injected into the mold, which is opened by an embossing gap. Finally, the material is molded to the plate geometry by a speed- and force-controlled compression stroke up to the compression forces in order to be investigated. After an isobaric and isothermal holding phase, the mold is cooled to the demolding temperature while maintaining the cavity pressure constant.

For the investigation of the electrical conductivity, different compounds were processed using the same parameter settings. The main process parameters are listed in Table 2, with bold values indicating the mold temperature and compression force used. Since the surface layer has an electrically insulating effect, it has to be removed by means of a surface treatment. Within this study, oxygen plasma etching was applied to remove the electrically insulating surface layer uniformly and in a defined manner from the produced plates, which were used for measuring the electrical conductivity. For this purpose, an etching device type Tepla 440 (Technics Plasma GmbH, Kirchheim, Germany) was utilized to treat the plates for 5 × 5 min with 500 W power output.

To study the achievable plate aspect ratios and the stability of sample thickness in the ICM-DT process, compression force was varied and evaluated in three factor steps at a mold temperature of 170 °C. The same has been performed for three different mold temperatures at a constant compression force of 400 kN. These parameters were chosen based on the analytical process design. All other molding parameters were kept constant. In each case, five test specimens were produced per setting. An overview of the main process parameters for the investigation of achievable plate aspect ratios and the stability of sample thickness in the ICM-DT process is also shown in Table 2.

In order to consider a pressure-dependent solidification of the melt, the process sequence was additionally designed as multi-stage ICM-DT process. In a first compression stage with high compression speed (15 mm·s^−1^) and high compression force (400 kN), the replication of the plate aspect ratios should be ensured. In a pressure relief stage within 0.5 s to 10 kN compression force, the flowability of the polymer should be restored within the completely filled cavity. Afterwards, a second compression at a slow speed (1 mm·s^−1^) up to a lower compression force (100 kN) is applied. Thus, the necessary flow processes for material compensation can take place with moderate pressure buildup. The three-stage process sequence is shown in Figure 2b. The parameter setting at the different compression stages are shown in Table 3. The other parameters were the same as for the determination of the electrical conductivity which are listed in Table 2.

### 2.3. Thermogravimetric Analysis

ICM-DT experiments for investigating possible thickness dimensional deviations at high filler contents and minimum wall thickness were carried out with the compound with maximum filler content. This material has been selected due to the requirement of high electrical conductivity for the application field of bipolar plates. For this material, the actual filler content was determined by a thermogravimetric analysis in a TGA Q5000 (TA Instruments, New Castle, DE, USA) under nitrogen atmosphere because the greatest deviations are to be expected here in the metering process of compounding. The filler content was evaluated after the thermal decomposition of the polymer at a temperature of 500 °C. In addition, to ensure that no decomposition of the graphite started at this temperature or was distorted by carbonaceous residues of the PP, pure graphite powder and pure PP were measured accordingly.

### 2.4. Analytical Process Characterization

The crystallization of PP shows a strong pressure dependence [43]. Thus, the selection of a mold temperature above the crystallization temperature range for the ICM-DT experiments is not satisfactory to ensure that the polymer is still sufficiently flowable during the filling process via the compression stroke. To be able to evaluate and interpret the solidification behavior of the material in combination with the generated process data, the compound was characterized analytically with regard to its pressure-dependent crystallization behavior. For this purpose, the absolute position of the solidification range of the compound was first determined by means of differential scanning calorimetry (DSC) in a TMDSC Q1000 (TA Instruments, New Castle, DE, USA) at a cooling rate of 20 K·min^−1^. The pressure dependence of the solidification range was determined by pressure-volume-temperature (pvT) measurements in a Rheograph 25 (Goettfert Werkstoff Prüfmaschinen GmbH, Buchen, Germany) in the temperature range of 60–220 °C at pressure levels of 200, 400, 800, 1200, and 1600 bar. The linear regression of the crystallization onset temperature as a function of pressure in the pvT measurements in combination with the absolute position of the crystallization peak from the DSC measurements allowed the calculation of the pressure-dependent shift of the crystallization range. This methodology enabled the evaluation of the measured pressures and temperatures during the ICM-DT process with respect to the solidification behavior of the compound. With this knowledge, whether a pressure or temperature induced solidification sets during the process can be investigated.

### 2.5. Electrical Conductivity

**Figure 3:**
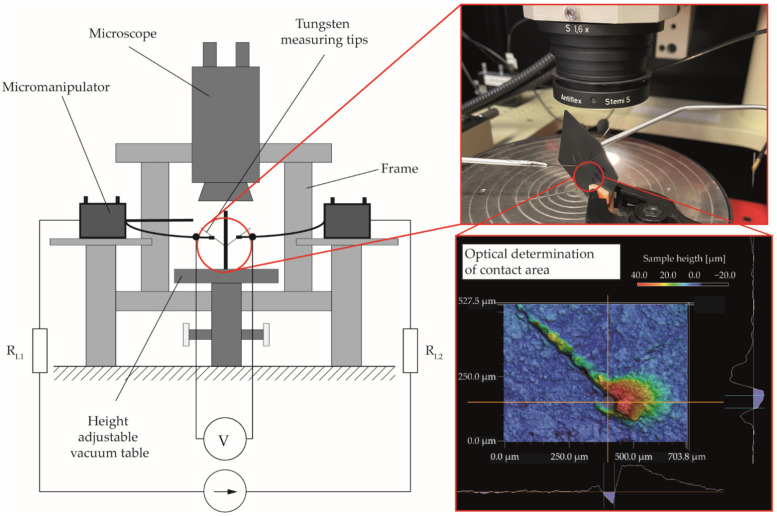
Schematic illustration of the measurement of the electrical resistance of the manufactured plate specimens.

First, the effects of the surface treatment were documented by microscopic images taken in the secondary electron detector of a scanning electron microscope type Ultra Plus (Carl Zeiss Microscopy GmbH, Jena, Germany) at an accelerating voltage of 10 kV under 1500-fold magnification. Afterwards, the electrical resistance of the plates was characterized by means of contacting via two tungsten manipulator tips (tip radius 7 µm) in the current range of −1000–1000 mA in a Precision Semiconductor Analyser 4156C (Agilent Technologies, Santa Clara, CA, USA), shown in Figure 3. To avoid the falsification of the results by co-determination of the line resistances *R_L1_* and *R_L2_*, the measurement was performed in a four-wire circuit. By evaluating the resulting voltage, the ohmic resistance *R* of the samples was determined via the derivative of the voltage–current curve. To determine the specific resistivity or the specific conductivity, the real cross-sectional area *A* of the current passage in the plate was determined by measuring the contacting point of the measuring tip using a laser scanning microscope type VK-X1000 (Keyence Corporation, Osaka, Japan) with a 20-fold magnification and the laser measuring mode, Figure 3.

To obtain the same contacting force and measuring position between the measuring tips for each plate, they were first set to the exact same opposite position without plates via the micromanipulators and the microscope. This resulted in the formation of furrows and material buildup on the sample surfaces when measuring with the plates between the measuring tips, since the needle is first pulled a short distance over the sample surface before the exact opposite position is reached. However, the actual contact point was always circular in shape and could, therefore, be easily detected by the optical evaluation software. By determining plate thickness *d* at the contacting point by means of an external micrometer, specific resistivity *ρ* and specific conductivity *σ* of the different compounds were determined using Equations (1) and (2), respectively.

(1) $$\rho =R*\frac{A}{d}$$

(2) $$\sigma =\frac{1}{\rho }=\frac{1}{R}*\frac{d}{A}$$

### 2.6. Plate Aspect Ratio and Dimensional Accuracy

**Figure 4:**
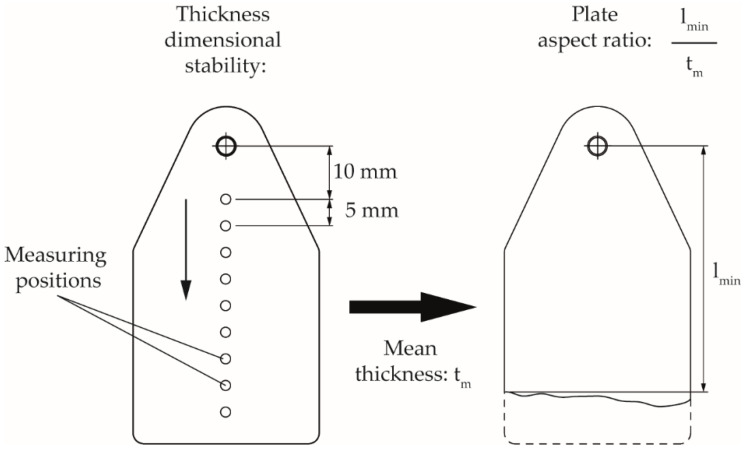
Measurement points for the investigation of the thickness dimensional stability and the plate aspect ratio.

The aspect ratio of the plate specimens produced under variation of the process parameters was calculated from the quotient of the minimal achieved flow path length (measured with a caliper gauge from the gate pin) and the average thickness of the plate specimens over the flow path, which are both shown in Figure 4. The thickness dimensional accuracy was evaluated using 10 measuring points with the outside micrometer along the flow path of the specimens, each at a distance of 5 mm. The measured thicknesses were normalized to the initial measuring point at the gate pin.

## 3. Results and Discussion

### 3.1. Filling Content

**Figure 5:**
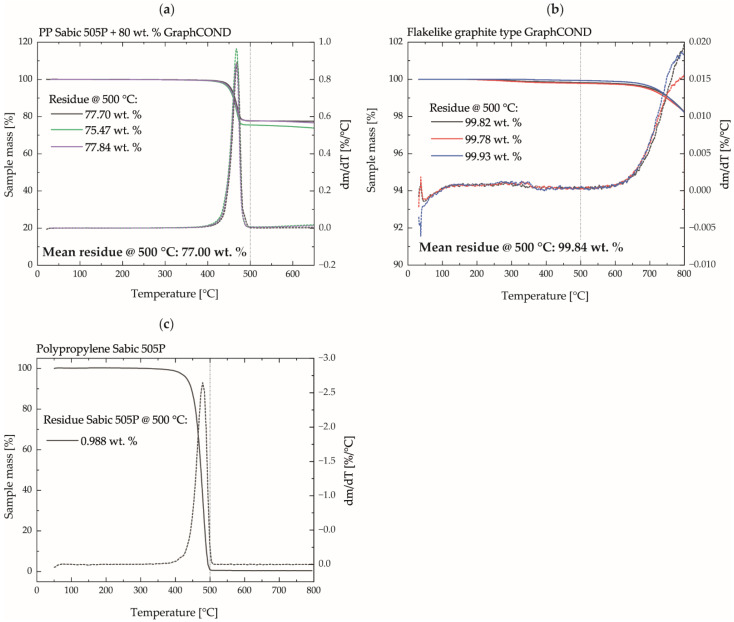
(**a**) Thermogravimetric analysis of the compound with maximum graphite content; (**b**) thermogravimetric analysis of the pure Graphite particles; (**c**) thermogravimetric analysis of the pure PP.

Figure 5 shows the results of the thermogravimetric analysis of the compound with a maximum calculated filler content of 80 wt.%. The measurement of the compound shows a clear decrease in mass from approx. 400 °C, which is completed at approx. 500 °C. The average residual mass of the compound determined at 500 °C $m_{c,res}\left(500\;{}^{\circ}C\right)$ is 77.0 wt.%, Figure 5a.

The results of the measurement of pure graphite show that there is hardly any decrease in mass at the measured temperatures. At 500 °C, a residual mass $m_{g,\;res}\left(500\;{}^{\circ}C\right)$ of about 99.8 wt.% graphite was evaluated, Figure 5b. The measurement error due to carbonaceous residues from the degraded PP was determined by measuring the pure PP and determining the residual PP mass content at 500 °C $m_{pp,res}\left(500\;{}^{\circ}C\right)\;$ to approx. 0.98 wt.%, Figure 5c. Thus, the resulting filler content in the compound $f_{c}$ used for the ICM-DT experiments is calculated according to Equation (3) to 75.8 wt.% and is in good agreement with the set filler content from the compounding.

(3) $$f_{c}=m_{c,\;res}\left(500\;{}^{\circ}C\right)-m_{pp,\;res}\left(500\;{}^{\circ}C\right)-\left(100-m_{g,res}\left(500\;{}^{\circ}C\right)\right)$$

### 3.2. Analytical Process Characterization

**Figure 6:**
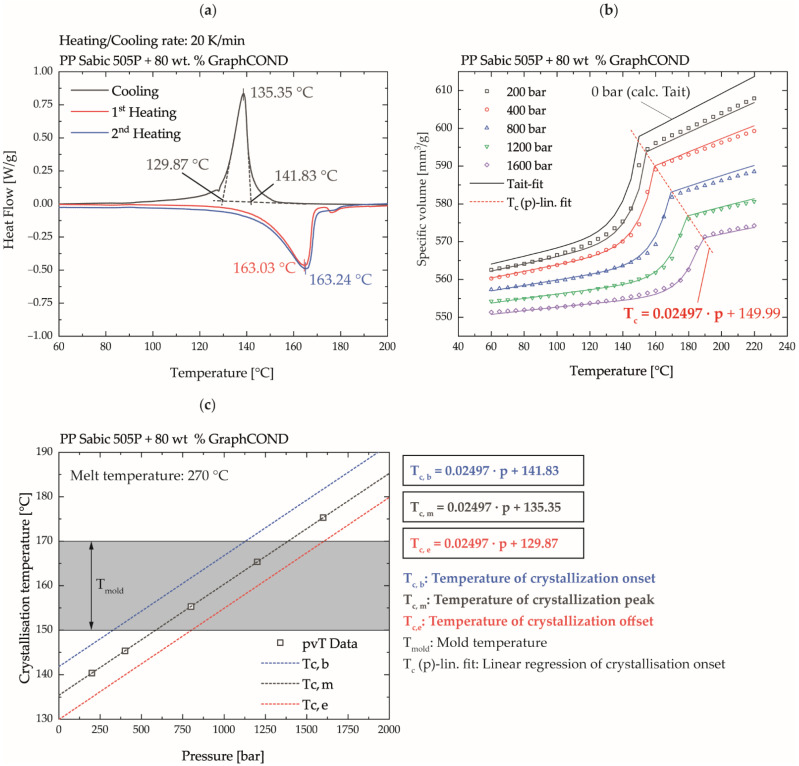
(**a**) Determination of the absolute position of the crystallization range based on DSC measurements; (**b**) determination of the pressure dependence of the crystallization range based on pvT measurements; (**c**) combination of the pressure dependence of the crystallization range from the pvT measurements and the absolute position of the crystallization range of the DSC measurements to determine the pressure- and temperature-dependent process window.

The results of the derivation of the pressure dependent crystallization range of the compound based on DSC and pvT measurements are shown in Figure 6. As displayed in Figure 6a, a crystallization onset temperature of approx. 142 °C, a crystallization peak temperature of approx. 135 °C, and a crystallization offset temperature of approx. 130 °C were measured. From the pvT investigations, the pressure dependence of the crystallization could be determined by a linear regression of the crystallization onset temperature, Figure 6b. The absolute position of the crystallization range of the pvT measurements at 0 bar pressure can only be calculated with the 2-domain Tait model and is distorted by the slow cooling rate of 2 K·min^−1^. For this reason, the equations for calculating the pressure-dependent shift of the crystallization range were derived from the determined slope of the pressure-dependent crystallization temperature of the pvT measurements and the determined y-intercepts as the absolute position of the solidification range of the DSC measurements, Figure 6c.

Accordingly, at the selected mold temperatures of 150, 160, and 170 °C, the pressure-temperature range shown in Figure 6c above the dashed straight lines does not yet result in any pressure-dependent solidification of the material. Thus, even if the melt is completely cooled to mold temperature during the injection process, it is ensured that pressures of up to 1600 bar can be applied at a mold temperature of 170 °C. At a mold temperature of 150 °C, pressures of approx. 750 bar can be applied to mold the high plate aspect ratio without complete pressure-induced crystallization of the material.

### 3.3. Electrical Conductivity

**Figure 7:**
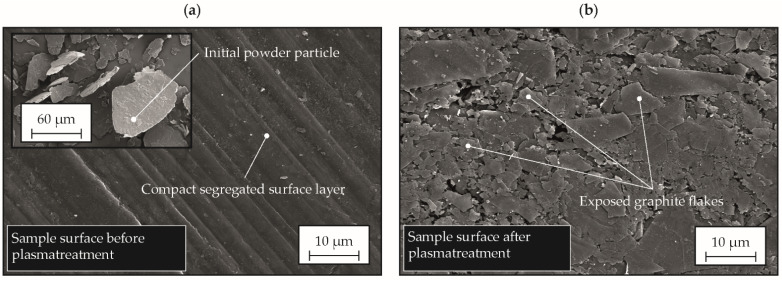
Scanning electron microscope images of the untreated (**a**) and the plasma-treated (**b**) sample surface.

The results of plasma etching, necessary for characterizing the electrical conductivity of the compounds, are exemplified by the compound with a maximum filler content of 75.8 wt.% in Figure 7. The picture of the untreated sample in Figure 7a shows that a compact and segregated polymer layer is formed on the surface of the samples.

The fountain flow within the injection molding cavity initially leads to an alignment of the fillers perpendicular to the flow direction at the flow front and in the boundary layer to the mold wall. Within the subjacent shear zone, they are then oriented in the flow direction, resulting in an almost complete segregation of the particles in the rapidly solidified boundary layer [44]. Investigations on the microstructure formation in the surface layers of unfilled PP as a function of mold temperatures have shown that even when the mold is tempered in the crystallization temperature range of the polymer, a fine crystalline to almost optically amorphous microstructure is formed in the surface region of the samples [45]. Figure 7b shows that the rapidly solidified boundary layer with this type of microstructure can be effectively removed by surface treatment using plasma and, thus, effectively expose the electrically conductive fillers. Thus, after plasma treatment, the graphite particles are exposed on the surface, making it much easier to couple the electrical current into the plate. For an industrial scale, a faster removal of the rapidly solidified boundary layer is required. Therefore, laser ablation could be a suitable method.

**Figure 8:**
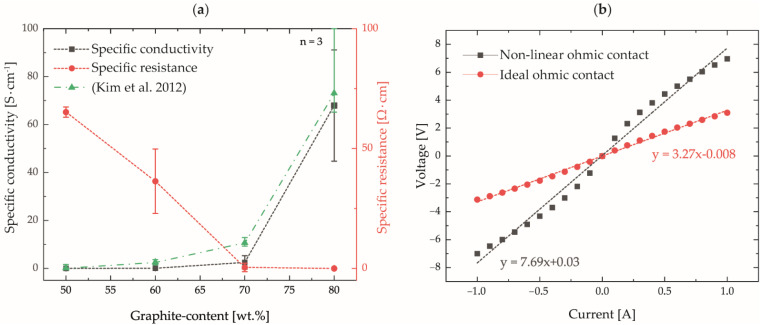
(**a**) Determined specific resistance and specific conductivities of the compounds produced in comparison with literature values [16]; (**b**) visualization of ideal and non-ideal contacting based on exemplary voltage–current curves.

The determination of the specific conductivity and specific resistance of the different compounds as a function of the graphite content is shown in Figure 8a. The measurements show an expected decrease in resistivity with increasing graphite content and an increase in electrical conductivity.

A comparison to the resistivity ranges of 10^12^–10^16^ Ω·cm provided in literature for pure polymers without fillers [19] shows that the percolation threshold is already reached with a filler content of 50 wt.%. However, high conductivities, as required for bipolar plate applications, are only achieved with filler contents of up to 80 wt.%. Since pure polymer can be considered a technical insulator due to its high resistivity values, the increase in electrical conductivity can be attributed exclusively to the increase in filler content. A filler content exceeding the pure percolation threshold leads to an increased contact probability of adjacent filler particles and, thus, to the formation of additional conducting paths along which the electrical current can flow. These results are in good agreement with investigations on the influence of the graphite content on the electrical conductivity of injection molded PPS plates (dimensions 60 × 60 × 2 mm^3^), which are also shown in Figure 8a [16].

Nevertheless, at maximum filler content, high standard deviations were measured for the specific conductivity and specific resistivity. This could be attributed to a falsification of the measurement results due to a non-ideal ohmic contact between the measuring tips and the plates. Due to the possible contacting of graphite particles that are not ideally connected to the percolation network or particles with a thin insulating residual polymer layer, a contact with non-linear resistivity behavior can form according to Li et al. [46] instead of an ideal ohmic contact, Figure 8b. The evaluation of the resistance as the slope of the linear regression model of the voltage–current curves and the contact over the small measurement tip radius led to significant measurement scatter. The optimization of the determination of the electrical properties by means of a special four-pole measuring electrode is, therefore, the subject of current investigations.

### 3.4. Plate Aspect Ratio and Dimensional Accuracy

**Figure 9:**
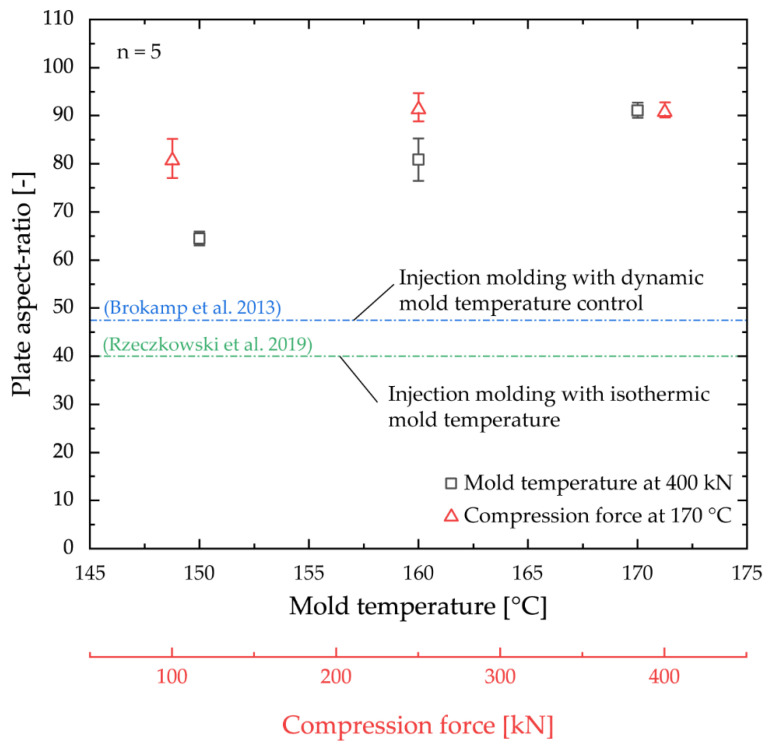
Molded plate aspect ratio as a function of mold temperature and set compression force compared with literature values [18,47].

Figure 9 shows a comparison of the molded plate aspect ratio with literature values for the production of highly filled bipolar plates. It can be seen that significantly higher plate aspect ratios can be achieved using the ICM-DT process.

The calculated plate aspect ratios in literature refer to significantly thicker plate geometries. For example, in studies on the processing of a PP graphite compound filled up to 80 wt.% into bipolar plates, the limits of injection molding processing were already reached with plate dimensions of 80 × 80 × 2 mm^3^ or an aspect ratio of 40 [18]. An older investigation on the molding accuracy of highly filled PPS graphite compounds (filler content 75 wt.%) using an injection molding process with dynamic mold temperature control (IM-DT) achieved slightly higher plate aspect ratios of ~48 with plate dimensions of 142.5 × 80 × 3 mm^3^ [47]. In the course of the present investigations, it was also tested whether the plates could be molded using a conventional injection molding and IM-DT process with the same mold, whereby only the embossing step was removed from the process sequence and the mold was completely closed at the start of the cycle. However, in this study, the production of completely filled plates according to this process sequence was not possible with any adjustment from mold temperature, injection speed, melt temperature, and holding pressure. In contrast, the molded aspect ratio using ICM-DT with the best setting (170 °C, 400 kN) could be increased by up to 125% compared to isothermal injection molding in [18] and by up to 90 % compared to IM-DT in [47]. These values represent minimum values since the plates were completely filled at a mold temperature of 170 °C and a compression force of 250 kN. The standard deviation of the data points when the mold is completely filled refers to slightly different thickness dimensions of the plates. Thus, the maximum possible plate aspect ratio in ICM-DT could not be identified and is, therefore, the subject of future investigations at the Institute of Polymer Technology. In this context, high compression forces and high mold temperatures favor the replication quality. However, if the compression force is set too low at 100 kN or mold temperatures < 170 °C, the mold’s cavity is not sufficiently replicated. It can be concluded that using the ICM-DT process significantly increased flow path lengths to wall thickness ratios can be achieved, particularly in the area of thin-walled components, despite high filling contents.

**Figure 10:**
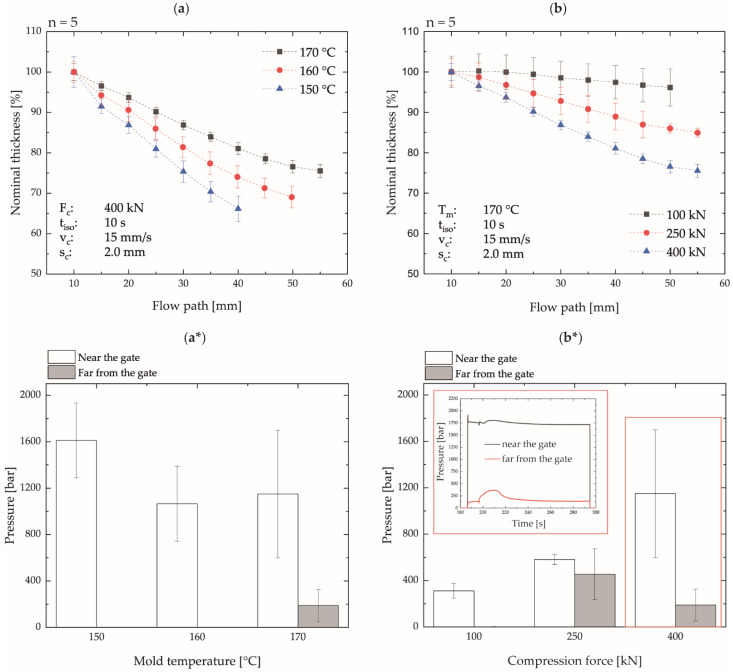
Nominal thickness dimensional stability over the flow path as a function of mold temperature (**a**) and compression force (**b**) as well as the associated maximum cavity pressures during compression at the positions near and far from the gate (a*,b*); abbreviations, see Table 2.

Even though the processing of highly filled materials into thin-walled planar components such as bipolar plates appears possible with the ICM-DT process, the evaluation of the thickness dimensional stability shows significant dimensional deviations over the flow path length. Here, low mold temperatures and high compression forces resulted in the greatest deviations within the plate, as displayed in Figure 10a,b.

The evaluation of the pressure signals in the mold at the positions near and far from the gate at the time of compression shows a significant pressure difference between the two measuring positions. This state is also persisted during the isobaric and isothermal holding phase and during the cooling phase, as shown in Figure 10a*,b*. For this reason, the compression force instead of the pressure signal is depicted in Figure 10. Due to the pressure-induced flow that underlies injection molding and injection compression molding, this difference would normally have to be compensated by flowing material. The magnitude of the pressure difference increases analogously to the thickness deviations with decreasing mold temperature and increasing compression force. Therefore, it can be assumed that the reduction in the flow cross-section during the compression step and the simultaneous increase in the surface-to-volume ratio leads to the faster cooling of the compound and a significant increase in pressure in the gating area. Since pressure decreases continuously towards the end of the flow path, the material can still flow at the end of the plate, but no more material can be fed to the end of the flow path from the areas near the gate. If the maximum pressures measured near the gate are compared with the analytical determination of the pressure-dependent solidification range of the compound, it becomes clear that, at a mold temperature of 170 °C and a pressure of about 1000 bar, the pressure-dependent onset temperature of crystallization is already reached. Only at the lower compression forces of 100 kN and 250 kN, this pressure undershot in the area close to the gate so that better thickness equalization can take place within the mold by flowing of the material. Thus, the plates produced with the lowest compression force showed insufficient molding of the cavity but, at the same time, the highest dimensional stability with respect to thickness. With mold temperatures of 160 and 150 °C, the solidification of the material already starts at lower pressures, which is why significantly greater thickness deviations have been be measured.

**Figure 11:**
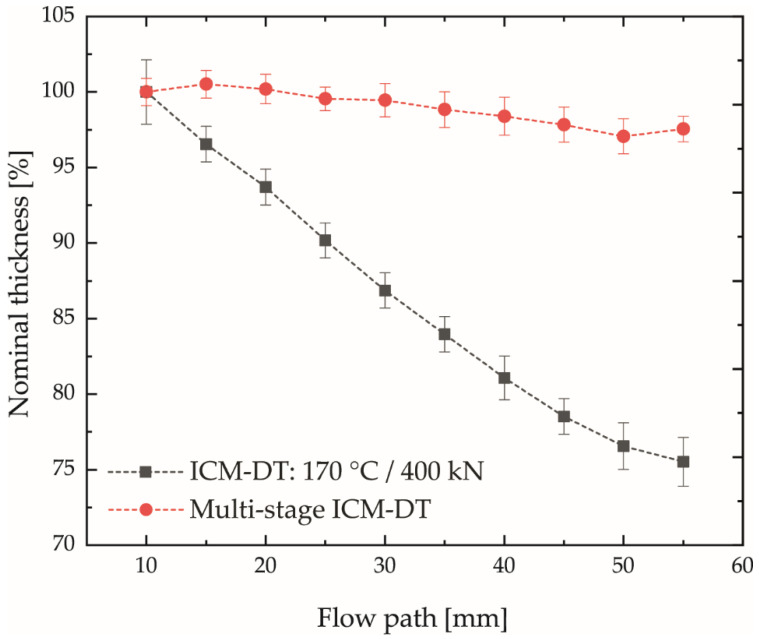
Achieved thickness dimensional stability of the multistage ICM-DT (see Figure 2b/Table 3) process compared to the single-stage process with maximum compression force and maximum mold temperature.

In contrast, the results of the multistage process show a significantly improved thickness dimensional accuracy with simultaneously high molding accuracy of the plates. The determined plate aspect ratio of the multistage ICM-DT process was 91.3 ± 0.9 and, thus, corresponds to the achieved maximum value of the single-stage ICM-DT process (91.7 ± 2.8). The achieved thickness deviation of the multistage process reached a maximum of 3% over the flow path in comparison to 20% at the single-stage ICM-DT at maximum aspect ratio and is shown in Figure 11. The results illustrate that the multistage ICM-DT process made it possible to circumvent the pressure-induced solidification of the melt in the single-stage process. Thus, the first compression stage with high compression speed and high compression force initially allowed the plate to be completely molded. The second pressure relief stage restored the flowability of the compound at the high mold temperature so that material equalization within the plate could be achieved by the third compression stage with low compression speed and compression force. Accordingly, an adapted multi-stage ICM-DT process enables the processing of highly filled compounds into thin-walled planar bipolar plates with high thickness dimensional stability and high flow path lengths to wall thickness ratios. The extent to which this process can be used to replicate fine channel structures with high aspect ratios is the subject of current research activities.

## 4. Conclusions

In this study, an injection-compression molding process with dynamic mold temperature control (ICM-DT) was investigated with regard to its ability to produce plate specimens (as an example for PEMFC bipolar plates) with high dimensional stability and high flow path length to wall thickness ratio (plate aspect ratio) from a highly filled polypropylene–graphite compound. For this purpose, four different compounds with different filler contents were prepared and investigated with respect to their electrical conductivity. The compound with maximum conductivity was characterized regarding its true filler content (75.8 wt.%) and the pressure-dependent solidification behavior. Additionally, it was investigated in the process by varying the mold temperature and the compression force with respect to achievable plate aspect ratios and thickness deviations over the flow path. The results of the ICM-DT experiments showed that high compression forces and high mold temperatures led to an increased plate aspect ratio by up to 125% compared to conventional processes from the current literature. It should be mentioned that the maximum possible aspect ratios in the ICM-DT process could not be investigated with the underlying plate specimen, since plate geometry can already be completely molded with certain parameter combinations. Moreover, the attempts to produce the present plate geometry with the highly filled compound in conventional injection molding or injection molding with dynamic mold temperature control were unsuccessful. Nevertheless, the plates in the ICM-DT process exhibited very high thickness deviations over the flow path. Here, low mold temperatures and high compression forces led to the greatest deviations. This effect could be attributed to a pressure-dependent solidification of the material in the gating area by means of the material characterization and the cavity pressure sensors. In order to eliminate this effect, a multi-stage process design was used in which the plate geometry is molded in a first compression stage with high compression force, high mold temperature, and high compression speed. In a second stage, the flowability of the melt is restored by decompression. In a subsequent third stage, the homogenization of the thickness dimensional stability is ensured by flow compensation processes with low compression speed and compression force. The multi-stage embossing process reduces the maximum deviation of the thickness over the flow path from approximately 20% to only 3% over the flow path, while keeping the maximum achieved plate aspect ratio with approximately 91 unchanged, compared to the single-stage process.

## Figures and Tables

**Table 1:** Main compounding parameters of the various compound materials.

Calculated Graphite Content (wt.%)	Dosing Speed PP Sabic 505 P (kg·h^−1^)	Dosing Speed GraphCOND (kg·h^−1^)	Nozzle Temperature (°C)	Screw Speed (min^−1^)
50	2.5	2.5	270	150
60	2.0	3.0		
70	1.5	3.5		
80	1.0	4.0		

**Table 2:** Main process parameters of the production of the plate specimens for the determination of the electrical conductivity (bold values) and the achievable plate aspect ratio as well as thickness dimensional accuracy.

Parameter (Unit)	Value
Mold temperature T_m_ (°C)	150/160/**170**
Heating time t_h_ (s)	180
Melt temperature T_M_ (°C)	270
Compression gap s_c_ (mm)	2.0
Injection flowrate v_i_ (cm^3^·s^−1^)	25.4
Compression speed v_c_ (mm·s^−1^)	15.0
Compression force F_c_ (kN)	100/250/**400**
Isothermal und isobaric compression time t_iso_ (s)	10
Cooling time t_cool_ (s)	80
Demolding temperature T_d_ (°C)	60

**Table 3:** Parameter setting of the multi-stage ICM-DT at the different compression stages.

Parameter (SI-Unit)	Value
Compression speed stage 1 v_c1_ (mm·s^−1^) (t_c1_)	15.0
Compression force stage 1 F_c1_ (kN)	400
Isothermal und isobaric compression time stage 1 t_iso1_ (s)	5
Compression speed stage 2 v_c2_ (mm·s^−1^) (t_c2_)	15.0
Compression force stage 2 F_c1_ (kN)	10
Isothermal und isobaric compression time stage 1 t_iso2_ (s)	5
Compression speed stage 3 v_c3_ (mm·s^−1^) (t_c3_)	1.0
Compression force stage 3 F_c3_ (kN)	100
Isothermal und isobaric compression time stage 3 t_iso3_ (s)	10

## Data Availability

The data presented in this study are available upon request from the corresponding author.

## Abbreviation

PEMFC	Polymer electrolyte membrane fuel cell
CNT	Carbon nanotubes
PPS	Polyphenylene sulfide
PP	Polypropylene
PA6	Polyamide 6
S/V	Surface to Volume ratio
ICM-DT	Injection compression molding with dynamic mold temperature control
IM-DT	Injection molding with dynamic mold temperature control
DSC	Dynamic differential calorimetry
pvT	Pressure–volume–temperature measurement
R	Electrical resistance
R_L,x_	Line resistance
A	Cross sectional area of the current passage
d	Plate thickness
ρ	Specific resistivity
σ	Specific conductivity
T_m_	Mold temperature
T_d_	Demolding temperature
T_c_	Crystallization temperature
T_c,b_	Temperature of crystallization onset
T_c,m_	Temperature of crystallization peak
T_c,e_	Temperature of crystallization offset
F_c,x_	Compression force in stage x
v_c,x_	Compression speed in stage x
T_iso,x_	Isothermal and isobaric compression time in stage x
t_h_	Heating time
T_M_	Melt temperature
s_c_	Compression gap
v_i_	Injection flowrate
t_cool_	Cooling time
f_c_	Filling content
m_c/pp/g,res_	Residual mass of the compound/polypropylene/graphite
